# Microbiological Safety of Donor Human Milk: Comparing Culture-Based Methods for Enterobacterales Detection

**DOI:** 10.3390/microorganisms13102259

**Published:** 2025-09-26

**Authors:** Lena Dawczynski, Nora Helke Leder, Sabine Trommer, Frank Kipp, Claudia Stein

**Affiliations:** Institute for Infectious Diseases and Infection Control, Jena University Hospital, 07747 Jena, Germany; lena-d@mail.de (L.D.); nora.leder@med.uni-jena.de (N.H.L.); sabine.trommer@med.uni-jena.de (S.T.); frank.kipp@med.uni-jena.de (F.K.)

**Keywords:** donor human milk, microbiological screening, culture-based methods, Enterobacterales, neonatal care, neonatal feeding, diagnostic sensitivity, unpasteurised milk

## Abstract

In neonatal care, donor human milk (DHM) is used when maternal milk is unavailable or insufficient. In several countries, including Germany, raw (i.e., unpasteurised) DHM is occasionally administered under specific clinical conditions. However, the lack of standardised, evidence-based microbiological testing protocols raises concerns about the reliability of safety assessments for this high-risk patient group. The objective of this study was to assess the performance of four culture-based microbiological methods for detecting Enterobacterales in donor human milk, using both spiked samples and raw milk. We compared the detection limits of four culture-based microbiological methods, with and without enrichment, using spiked DHM samples and 93 raw DHM samples from a single donor (limited generalisation). Artificially inoculated samples contained defined concentrations of *E. coli*, *K. pneumoniae*, and *S. ureilytica*. Detection limits varied by several orders of magnitude (2.86 × 10^2^ CFU/mL to 4.90 × 10^0^ CFU/mL). In real samples, enrichment-based methods detected Gram-negative pathogens in four out of ninety-three samples (three *S. ureilytica*, one *P. juntendi*); direct plating detected none. Increasing the sample volume and applying enrichment improved detection sensitivity. Whole-genome sequencing confirmed species identity and showed that the *S. ureilytica* isolates from a single donor were clonally related, indicating a recurring detection pattern and underscoring the need for longitudinal microbiological monitoring. In view of the new EU SoHO Regulation classifying DHM as a Substance of Human Origin, these findings highlight the urgent need for standardised, sensitive protocols to ensure neonatal safety.

## 1. Introduction

A mother’s own milk remains the optimal source of nutrition for preterm infants. When unavailable or insufficient, donor human milk (DHM) is the recommended alternative, offering clear advantages over formula [1,2]. Donor human milk offers several advantages over infant formula, most notably a reduced incidence of necrotising enterocolitis [3,4]. Furthermore, limited evidence suggests additional protective effects of human milk in reducing the incidence of sepsis, bronchopulmonary dysplasia, and retinopathy of prematurity [3,5,6]. In the longer term, human milk is also associated with improved neurodevelopmental and metabolic outcomes, including a reduced risk of obesity, and diabetes [7,8].

A key distinction, however, lies in whether DHM is administered raw or pasteurised. International guidelines, including those from the European Milk Bank Association (EMBA); European Society for Paediatric Gastroenterology, Hepatology and Nutrition (ESPGHAN); American Academy of Pediatrics (AAP); and Human Milk Banking Association of North America (HMBANA), advocate for pasteurisation, primarily due to its efficacy in inactivating a wide range of pathogens, including cytomegalovirus (CMV) [9,10,11,12,13,14]. However, pasteurisation can alter the nutritional and bioactive composition of human milk [15]. Most European countries rely almost exclusively on pasteurised DHM, in accordance with the recommendations of the EMBA [2,16]. Germany and Norway, however, occupy a unique position, as they also permit the use of raw donor human milk under specific conditions [2,17]. In Germany, raw DHM may be administered if the donor tests negative for CMV-IgG and -IgM, with quarterly reassessments, and if batch-specific microbiological screening is conducted. The milk must be discarded if *Bacillus cereus* is detected or if donors are colonised with organisms such as Methicillin-resistant *Staphylococcus aureus* (MRSA), multidrug-resistant Gram-negative bacteria (3- or 4-MRGN), or Vancomycin-resistant Enterococci (VRE) [2]. Despite these procedural requirements, the current German clinical guideline does not provide evidence-based recommendations on the use of raw DHM.

Despite these precautions, raw DHM is not sterile, and microbial contamination remains a concern [18,19,20]. The most frequently detected microorganisms are coagulase-negative staphylococci (CoNS), which are part of the normal skin microbiota but may act as opportunistic pathogens—particularly in preterm or immunocompromised infants [21]. Of greater concern is the presence of pathogenic Gram-negative bacteria, including members of the order Enterobacterales, which pose a significantly higher risk of causing severe systemic infections. Several cases of breast milk-associated infections have been documented in the literature, involving pathogens such as *Escherichia coli*, *Klebsiella pneumoniae*, and *Serratia marcescens* [18,19,20].

Currently, there are no standardised, evidence-based guidelines for evaluating bacterial contamination in donor human milk. However, historically established recommendations and threshold values serve as reference points for guidance [2]. These guidelines provide only threshold values and do not include procedural instructions for the microbiological examination of raw DHM. To date, a universally standardised approach is lacking, which is crucial for ensuring a reliable microbiological assessment and the safety of preterm infants [2,22].

In Germany, DHM is categorised in clinical practice based on total bacterial count and the presence of specific pathogens. DHM containing ≤10^3^ colony-forming units per mL (CFU/mL) may be administered fresh and unpasteurised to preterm infants weighing less than 1500 g. DHM with bacterial counts between 10^3^ and 10^4^ CFU/mL—typically comprising skin commensals—is stored at −20 °C and may be used without pasteurisation for preterm infants over 1500 g or for older infants. Milk containing 10^4^ to 10^5^ CFU/mL is subjected to Holder pasteurisation (62.5 °C for 30 min), provided the count of potentially pathogenic bacteria such as *Staphylococcus aureus*, Gram-negative rods, Streptococcus groups A and B, or *Pseudomonas aeruginosa* remains at or below 10^5^ CFU/mL. If this threshold is exceeded, the milk is discarded [2].

Data from a retrospective case–control study indicate that even low-level contamination of raw donor human milk—specifically with *Serratia marcescens* at concentrations below 10^2^ CFU/mL—can lead to colonisation and invasive infection in preterm infants. In one reported case, *S. marcescens* was cultured from both cerebrospinal fluid and blood of a neonate who subsequently developed severe brain damage and died, despite immediate antibiotic therapy. [18]. This highlights the need for more sensitive and standardised culture-based methods to reliably assess the microbiological quality of raw DHM.

In this context, the present study aimed to systematically compare four culture-based detection methods for Enterobacterales—specifically *Serratia ureilytica*, *Klebsiella pneumoniae*, and *Escherichia coli*. A key focus was the determination of detection limits to assess the reliability of each method in identifying Enterobacterales in DHM. To enhance clinical relevance, all methods were additionally assessed for their practical applicability using real-life donor milk samples.

## 2. Materials and Methods

### 2.1. Comparison of Cultural Methods for the Detection of Enterobacterales in Spiked Milk Samples

This study aimed to compare four culture-based methods for detecting Enterobacterales. To evaluate their effectiveness, milk samples were deliberately spiked with clinical *Serratia ureilytica* isolate (strain ID: S.35.25.Sm), *Klebsiella pneumoniae* ATCC 700603, and *Escherichia coli* strain ATCC 25922. *E. coli* ATCC 25922 and *K. pneumoniae* ATCC 700603 are widely used for quality control purposes, while *S. ureilytica* S.35.25.Sm was chosen as a clinically relevant representative of the *Serratia* genus based on recent detection in raw donor milk. Whole-genome sequencing confirmed species identity and did not detect any acquired antibiotic resistance genes in this isolate.

For each bacterial strain (*S. ureilytica*, *K. pneumoniae*, and *E. coli*), serial tenfold dilutions ranging from 10^8^ to 10^0^ CFU/mL were prepared. For each dilution, three independent biological replicates were generated on separate days. Each biological replicate was processed in duplicate (technical replicates), resulting in six measurements per dilution step and strain (*n* = 6). Each technical replicate was plated onto two agar plates to allow for accurate colony enumeration. This experimental design was identically applied across all four tested culture-based methods. The final cell count was determined by calculating the mean of the cell counts from the technical replicates. The samples were cultured on four distinct media, and the resulting colonies were enumerated to assess the relative efficiency of each method.

To test the detection limits under realistic conditions, we used pasteurised donor milk as carrier material for the spiking experiments. The milk had been obtained from voluntary donors, pasteurised, and stored frozen until use. The effectiveness of pasteurisation was verified by including culture-based negative controls. As the milk served solely as background material and no personal data were collected, ethical approval was not required.

### 2.2. Determination of Detection Limits for Cultural Methods Using Spiked Milk Samples

**Figure 1:**
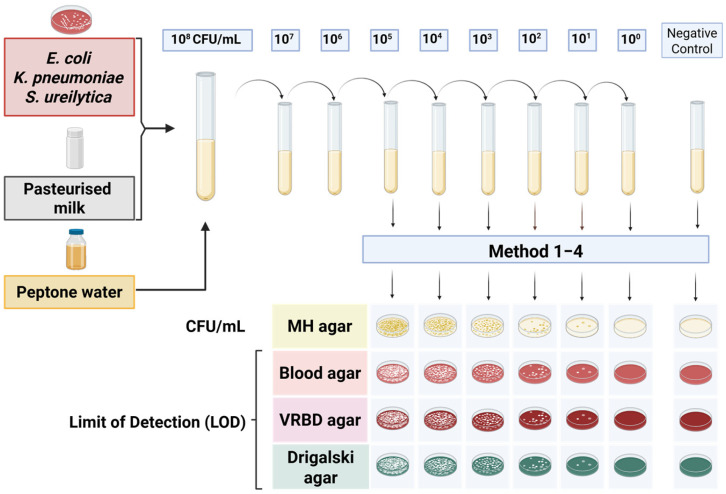
Overview of the experimental setup for determining detection limits in spiked samples. The serial dilution series was cultured on various agar media to assess colony counts and determine the lowest bacterial concentration at which colony growth was detectable.

To determine the detection limits, a dilution series of spiked samples was prepared, ranging from 10^8^ to 10^0^ CFU/mL. The diluted samples were cultured on various agar media: Müller–Hinton agar (Becton Dickinson GmbH, Heidelberg, Germany), Columbia blood agar (Becton Dickinson GmbH, Heidelberg, Germany), Drigalski lactose agar (Becton Dickinson GmbH, Heidelberg, Germany), and Violet Red Bile Dextrose (VRBD) agar (Merck, Darmstadt, Germany), to assess the colony counts and establish the limit of detection, defined as the lowest bacterial concentration at which colony growth was still observed. Of the media used, Müller–Hinton and Columbia blood agar are non-selective, Drigalski lactose agar is selective for Gram-negative bacteria, and VRBD agar is both selective and differential for Enterobacteriaceae. As part of each experimental run, negative controls consisting of uninoculated culture media and pasteurised donor milk were included in parallel with each biological and technical replicate to confirm sterility and exclude contamination. The experimental setup for the dilution series and agar plate inoculation is shown in Figure 1, illustrating the procedures for determining the detection thresholds.

### 2.3. Culture-Based Detection Methods

Four culture-based methods were employed to detect Enterobacterales in spiked DHM samples, each differing in terms of the volume of milk plated, the use of centrifugation, and pre-enrichment procedures.

**Figure 2:**
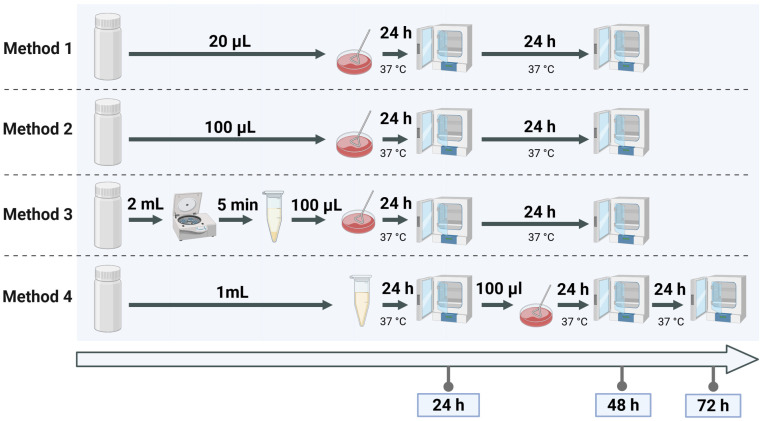
Visual representation of the four culture methods for detecting Enterobacterales in spiked samples. Method 1 and Method 2 differ by plating volume, Method 3 includes a centrifugation step, and Method 4 incorporates pre-incubation for culture enrichment.

Method 1, used as the reference, reflects the current routine protocol at the Jena University Hospital and involves direct plating of 20 µL of donor milk onto selective media. Method 2 increases the plated volume to 100 µL of donor milk to enhance sensitivity. Method 3 includes a centrifugation step to concentrate bacterial cells from 1 mL of donor milk before plating the pellet. Method 4 incorporates 24 h pre-incubation of 1 mL donor milk in buffered peptone water, based on a standardised method originally developed for the detection of Enterobacteriaceae in food matrices [23], adapted here for use in human milk screening. The experimental setup for these methods is depicted in Figure 2, which illustrates the step-by-step procedure.

### 2.4. Assessment of Method Performance on Raw Donor Milk

To evaluate the performance of four detection methods under conditions simulating routine diagnostic use, 93 raw donor milk samples were collected from a single donor between late October 2023 and February 2024. In September and early October 2023, Serratia species had been detected in several donor milk samples from this individual during routine microbiological screening. Due to a suspected epidemiological link between these milk samples and Serratia detections in neonatal patients (unpublished data), the donor was excluded from further milk provision. The corresponding bacterial isolates from September and early October were not available for this study. However, subsequently donated milk samples from late October onwards remained stored frozen and were made available for extended microbiological analysis to clarify the suspected transmission event from a hospital hygiene perspective. This unique clinical context enabled us to assess multiple raw milk samples from the same donor under controlled laboratory conditions. Due to constraints related to clinical supply and usage, no additional milk samples from other donors could be obtained for research purposes. While the use of samples from a single donor limits the generalisability of our findings, it allowed for a detailed longitudinal assessment of microbial presence and method performance over time. The milk was collected in a clinical setting and immediately frozen at −20 °C. Prior to microbiological analysis, all samples were completely thawed at room temperature. Columbia blood agar (BD, Heidelberg, Germany) was utilised for the detection of Gram-positive pathogens, while MacConkey agar (BD, Heidelberg, Germany) was used for the detection of Gram-negative pathogens. The results were recorded after 24 and 48 h of incubation at a temperature of 37 °C. For all Gram-negative detections, short-read sequencing was performed to confirm species identification.

### 2.5. Short-Read Sequencing

High-throughput WGS and subsequent data analysis were then performed with MiniSeq Illumina as described before [24]. Genomic DNA was paired-end-sequenced with a MiniSeq Reagent kit v2 150 bp (Illumina, San Diego, CA, USA) with an average insertion size of 300 bp. The resulting reads were quality-trimmed and de novo assembled using the Velvet algorithm integrated into Ridom SeqSphere+ software, version 10.0.3 (Ridom GmbH, Muenster, Germany). For *Serratia ureilytica* an ad hoc cgMLST was established according to Ridom SeqSphere+ guidelines we used public core-genome multilocus sequence typing (cgMLST) (reference genome: *Serratia marcescens* subsp. *marcescens* ATCC 13880 chromosome, complete genome). Minimum-spanning tree analysis was performed based on the determined allelic profiles using the Ridom SeqSphere+ software, version 10.0.3 with the parameter “pairwise ignore missing values”. We defined a cluster of clonal isolates if the cgMLST revealed a difference of ≤10 alleles for *Serratia ureilytica*. The whole-genome sequencing data of the *Serratia ureilytica* isolates have been deposited in the European Nucleotide Archive (ENA) under project accession number PRJEB95798, with individual sample accession numbers ERS25447765 (S.216.24.Sm), ERS25447766 (S.218.24.Sm), and ERS25447768 (S.219.24.Sm).

### 2.6. Statistical Analysis

In preliminary experiments, four detection methods were evaluated. For each method, the mean ($\bar{x}$) and standard deviation (s) were calculated across replicates to allow for quantitative comparison. The mean ($\bar{x}$) was defined as

(1) $$\bar{x}=\frac{1}{n}\sum_{i=1}^{n}x_{i}$$

The standard deviation (s) was defined as

(2) $$s=\sqrt{\frac{1}{n-1}\sum_{i=1}^{n}{\left(x_{i}-\bar{x}\right)}^{2}}$$

## 3. Results

### 3.1. Enrichment Steps Enhance Pathogen Recovery by Several Orders of Magnitude

To assess the performance of various microbiological detection methods for Enterobacterales in DHM, artificially spiked milk samples were analysed using four distinct approaches. The primary aim was to determine the detection limits of each method and evaluate how different methodological factors influenced the recovery of pathogens. No significant differences in colony counts were observed between the tested agar media; thus, all four methods were comparable in terms of quantitative recovery of Enterobacterales from spiked milk samples.

**Figure 3:**
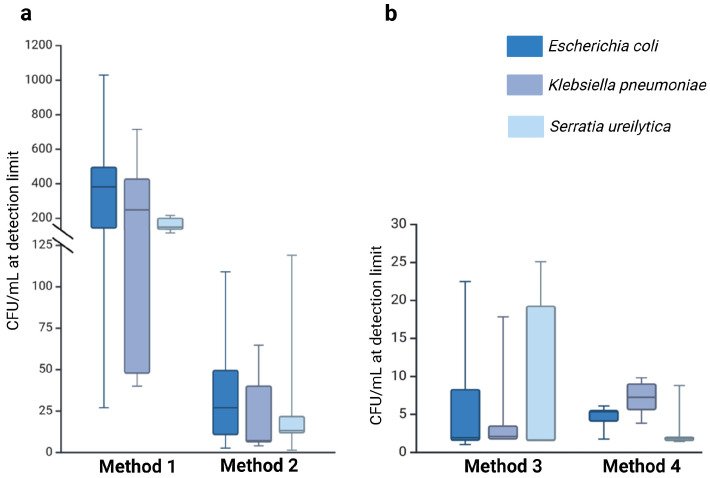
Detection limits (LOD) for *E. coli*, *K. pneumoniae*, and *S. ureilytica* across the four methods, illustrating the effects of sample volume and enrichment on pathogen recovery. Method 1 used a small sample volume, while Methods 2, 3, and 4 used a larger sample volume (100 μL). Methods 1 and 2 were performed without enrichment (**a**), whereas Methods 3 and 4 included an enrichment step (**b**). The coloured box represents the interquartile range (IQR), the horizontal line within the box indicates the median, and the whiskers extend to 1.5 × IQR, illustrating the data distribution.

Methods that included pre-analytical enrichment (Method 3: 6.74 × 10^0^ CFU/mL; Method 4: 4.90 × 10^0^ CFU/mL) exhibited significantly lower median detection limits compared to the direct plating methods (Method 1: 2.86 × 10^2^ CFU/mL; Method 2: 2.81 × 10^1^ CFU/mL). The detection limits varied by several orders of magnitude, highlighting the pivotal role of enrichment steps in enhancing pathogen recovery (Supplementary Materials Table S1). Additionally, the volume of milk sampled had a marked impact on detection efficiency. Methods employing larger volumes (100 µL; Methods 2–4) recovered pathogens more reliably than Method 1, which used only 20 µL. These findings highlight the critical role of both sample volume and methodological approach, with enrichment steps and increased input volumes substantially improving detection sensitivity. A summary of these findings, including the effect of sample volume and enrichment on pathogen recovery, is illustrated in Figure 3 below.

To assess the applicability of these methods to non-spiked samples, all four approaches were subsequently applied to 93 raw donor milk specimens.

### 3.2. Methodology Influences Detection of Gram-Negative Pathogens in Raw Milk

Ninety-three raw donor milk samples from a single voluntary donor were analysed to assess their microbial composition. All samples contained Gram-positive bacteria, predominantly coagulase-negative staphylococci. Bacterial counts were below 10^2^ CFU/mL in 49 samples, were between 10^2^ and 10^3^ CFU/mL in 37 samples, and exceeded 10^3^ CFU/mL in 7 samples. Gram-negative pathogens were identified in four of the ninety-three samples: three tested positive for *Serratia ureilytica* and one for *Pseudomonas juntendi*. Subsequent sequencing of the Gram-negative isolates validated the species-level identification.

**Figure 4:**
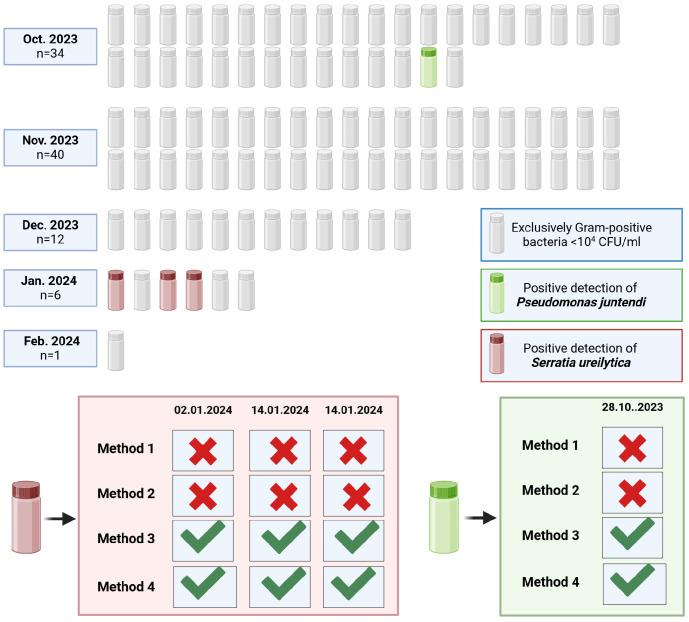
Overview of bacterial findings in 93 raw donor milk samples. Green check marks indicate successful pathogen detection, while red crosses signify the absence of detection. The months and dates reflect the period during which the milk was donated.

The detection of Gram-negative pathogens in raw donor milk was markedly influenced by the microbiological method applied. Only enrichment-based approaches (Methods 3 and 4) enabled the identification of *Serratia ureilytica* and *Pseudomonas juntendi*, whereas no Gram-negative organisms were recovered using direct plating methods (Methods 1 and 2). These findings demonstrate that pre-enrichment substantially enhances the recovery of low-abundance Gram-negative bacteria that may escape detection through direct culture (Figure 4).

### 3.3. Clonal Persistence of Serratia ureilytica in Repeated Donor Milk Samples

The molecular analyses confirmed all culture-based findings and provided additional confirmation of isolate identity. All three *S. ureilytica* detections originated from samples collected in January 2024. Comparative sequence analysis revealed a high degree of genetic similarity among these three isolates. Based on whole-genome relatedness, these Serratia strains were classified as clonal (Supplementary Materials Figure S1). In September and early October 2023, the same donor’s milk had already tested positive for *Serratia* spp. in routine diagnostics on multiple occasions. However, the corresponding isolates from that period were no longer available for sequencing. The confirmed clonality of the January isolates, together with earlier microbiological findings, suggests persistent colonisation or recurring contamination of the donor’s milk over time. These findings underscore the importance of continuous microbiological monitoring to ensure the consistent safety and quality of donor human milk.

## 4. Discussion

The present study compared various microbiological detection methods for Enterobacterales in DHM and assessed their diagnostic accuracy. To ensure applicability under real-world conditions, the methods were tested directly on donor milk samples. The findings demonstrated considerable variation in the diagnostic performance of the detection methods, with mean limits of detection ranging from 2.86 × 10^2^ CFU/mL to 4.90 × 10^0^ CFU/mL, indicating differences spanning several orders of magnitude. Due to the limited sample size, caution is required when extrapolating these results to larger populations.

This variability in detection thresholds directly influences the guideline-defined threshold values, which provide recommendations for the handling of raw DHM donations in clinical settings. These findings underscore the need for standardised microbiological testing protocols to ensure consistent and reliable quality assessments of human milk. Such standardisation would support informed decision-making regarding subsequent processing steps, including pasteurisation, disposal, or the use of raw DHM [2,9,22].

Enrichment techniques proved especially effective in enhancing the detection of Gram-negative bacteria. *Serratia ureilytica* and *Pseudomonas juntendi* were recovered exclusively through enrichment-based methods (Methods 3 and 4), but remained undetected by direct plating (Methods 1 and 2). Both organisms have been reported in raw donor milk in relation to nosocomial infections and outbreaks, and insufficient detection may pose a risk to vulnerable populations, particularly preterm infants [18,19,20].

Increasing the inoculum volume also significantly improved detection. Methods using 100 µL (Methods 2–4) consistently outperformed Method 1 (20 µL). These observations align with previous findings in veterinary diagnostics: Krömker et al. demonstrated that increasing the inoculum from 10 µL to 100 µL, alongside alternative detection strategies, enabled the recovery of pathogens in previously culture-negative bovine mastitis samples [25]. Similarly, Dinsmore et al. found that extended pre-incubation combined with higher inoculum volumes markedly improved detection rates in clinical mastitis [26].

Beyond sample volume and incubation, centrifugation (Method 3) also emerged as a valuable enrichment step. Previous studies have shown that centrifugation followed by resuspension can markedly enhance pathogen recovery from milk samples. At a bacterial concentration of 1 CFU/mL, centrifugation increased the detection rate to 75%, compared to only 18.75% without this step [27]. These findings highlight the effectiveness of centrifugation, particularly in samples with low bacterial loads, for improving the sensitivity of microbiological detection. Further research by Brewster et al. demonstrated that centrifugation significantly enhanced bacterial recovery from pasteurised cow’s milk, achieving an efficiency of 98% [28]. However, in raw milk, the recovery rate was only about 7%, as bacteria remained trapped in the cream layer. By introducing horizontal agitation for 10 min, recovery rates exceeded 95% [28]. A similar method also showed promising results in a separate study of human milk samples [29]. In the present study, cream layer formation was observed, but this was not further investigated, representing a limitation of the current study.

In conclusion, the choice of microbiological method substantially influences the detection of Gram-negative pathogens in donor milk. The observed variation between methods may help explain why infections with Enterobacterales can occur even at low bacterial counts, as reported by Bechmann et al. [18]. More sensitive approaches—particularly Methods 3 and 4—enabled the detection of pathogens such as *Serratia ureilytica* and *Pseudomonas juntendi*, which remained undetected with direct plating. As no universally accepted minimum infectious dose has been established for Enterobacterales in raw donor human milk, especially in the context of preterm neonates, the precautionary use of highly sensitive detection methods is warranted to minimise infection risks. However, recent outbreak investigations suggest that even very low bacterial concentrations—below 10^2^ CFU/mL—may be sufficient to cause colonisation or invasive infections in vulnerable infants, particularly when associated with opportunistic pathogens such as *Serratia marcescens*. These findings emphasise the need for a risk-based diagnostic approach that combines microbiological results with clinical assessment criteria. Future efforts should aim to define evidence-based thresholds and integrate them into guidelines for DHM screening and release.

The use of highly sensitive detection methods, such as Method 3 and Method 4, warrants critical evaluation with regard to their benefits and associated practical demands. While these approaches enable the identification of even minimal quantities of potentially pathogenic microorganisms, such findings do not necessarily imply clinical relevance [16,30]. This is partly due to the inherent antimicrobial properties of raw human milk [16,31], which may neutralise low-level contaminants. Furthermore, there is a lack of data regarding the minimum infectious dose required to trigger an infection, making it difficult to establish a clear threshold for clinical relevance. This raises the question of the practicality and necessity of highly sensitive detection methods in the context of human milk quality assurance.

A crucial factor to consider when selecting microbiological testing methods is their impact on turnaround time and laboratory workload. Pre-incubation-based enrichment protocols require an additional 18 to 24 h before plating, which substantially extends the total time needed for microbiological assessment. This may conflict with time-sensitive workflows in human milk banks: according to the current guidelines, thawed raw donor milk can be stored at 4 °C for a maximum of 48 h before administration to neonates. If microbiological testing is initiated only after thawing, enrichment protocols with delayed readout may risk exceeding this safety window. In contrast, centrifugation-based enrichment can be completed within minutes and seamlessly integrated into existing workflows without additional personnel or equipment. Thus, while both approaches increase diagnostic sensitivity, centrifugation offers a more resource-efficient and time-compatible alternative, particularly when timely clinical use of DHM is required.

However, the selection of detection methods must be carefully considered in light of their broader implications. The implementation of stricter microbiological regulations, particularly those based on sensitive detection techniques, may lead to increased rates of pasteurisation or disposal, potentially reducing the availability of DHM [16,32,33]. Nevertheless, a robust detection strategy is essential to accurately identify bacterial colonisation or contamination and minimise the risk of undetected pathogens. Striking a balance between microbiological safety and the sustained availability of DHM remains essential to ensure both clinical safety and equitable access.

One important limitation of this study is that all raw donor human milk (DHM) samples were obtained from a single donor. Whole-genome sequencing revealed that all *Serratia ureilytica* isolates detected in the 93 DHM samples were clonally related, indicating a recurring detection pattern suggestive of persistent contamination or colonisation.

While the single-donor approach enabled highly standardised conditions and minimised variability due to inter-individual factors, it also restricts the generalisability of our findings. The observed microbial patterns may reflect donor-specific dynamics not representative of the broader donor population. Variables such as the donor’s health status, lactation stage, or hygienic practices during milk collection may influence microbial profiles and detection sensitivity. To confirm and expand on our results, future studies should include a larger and more diverse cohort of donors across different clinical and organisational settings.

In addition to methodological and logistical aspects, forthcoming regulatory changes must also be taken into account. The new EU Regulation on Substances of Human Origin (SoHOs), adopted in 2024 and set to take effect in August 2027, classifies human milk as a SoHO for the first time [34]. Until now, human milk has been regulated as a food product in Germany under Regulation (EC) No. 178/2002 [35].

However, the revised legal framework raises its legal and regulatory status and aims to establish harmonised standards for processing, handling, and use to ensure the safety of donors and recipients.

The European Milk Bank Association (EMBA) is supporting this development through the IMAGINE-HMB project (Implementation of human Milk harmonized Guidelines for Infant Nutrition in Europe), which seeks to develop evidence-based guidelines for microbiological testing of DHM across the EU. In the long term, this initiative may contribute to the harmonisation of microbiological standards, thereby facilitating more efficient and safe use of DHM [36].

## 5. Conclusions

Our findings provide valuable insights for the development of optimal microbiological methods for the analysis of DHM. They demonstrate the critical importance of sensitive detection techniques for the reliable identification of potentially pathogenic bacteria, such as Enterobacterales. Given the vulnerability of neonates receiving DHM, standardised diagnostic procedures for assessing microbiological safety are essential. The integration and validation of such methods into routine diagnostics are crucial for establishing consistent detection standards and improving microbiological safety in the long term. Further research is needed to assess the applicability of the proposed methodology in various practical settings and to refine existing procedures.

## Data Availability

All data underlying the findings of this study are presented within the article. No additional deidentified participant data or supporting materials are available. The whole-genome sequencing data of the *Serratia ureilytica* isolates have been deposited in the European Nucleotide Archive (ENA) under project accession number PRJEB95798, with individual sample accession numbers ERS25447765 (S.216.24.Sm), ERS25447766 (S.218.24.Sm), and ERS25447768 (S.219.24.Sm). The authors take full responsibility for the integrity and accuracy of the data presented.

## Supplementary Materials

The following supporting information can be downloaded at: https://www.mdpi.com/article/10.3390/microorganisms13102259/s1, Table S1: This table presents the LOD values obtained for three Enterobacterales strains (*E. coli*, *K. pneumoniae*, and *S. ureilytica*) across four different culture-based methods. For each method, limit of detection was assessed using three independent biological replicates on separate days, each with two technical replicates. Each technical replicate was plated on two agar plates, and the mean colony count from these plates was used to determine the limit of detection (CFU/mL), as shown in the corresponding column. Figure S1: Minimum Spanning Tree illustrating the clonal relationships of three *Serratia ureilytica* isolates (S.216.24.Sm, S.218.24.Sm, and S.219.24.Sm) recovered from raw donor milk samples in January 2024. The numbers indicate the allelic differences based on core genome MLST (cgMLST), with all isolates differing by only one allele, indicating clonal relatedness.

## Abbreviations

The following abbreviations are used in this manuscript:

DHM	Donor human milk
EMBA	European Milk Bank Association
ESPGHAN	European Society for Paediatric Gastroenterology, Hepatology and Nutrition
AAP	American Academy of Pediatrics
HMBANA	Human Milk Banking Association of North America
CMV	Cytomegalovirus
MRSA	Methicillin-resistant *Staphylococcus aureus*
VRE	Vancomycin-resistant Enterococci
CoNS	Coagulase-negative Staphylococci
VRBD	Violet Red Bile Dextrose agar
cgMLST	Core-genome multilocus sequence typing
SoHO	Substance of Human Origin
LOD	Limit of detection
