# Supplementary material for: Microbiological Safety of Donor Human Milk: Comparing Culture-Based Methods for Enterobacterales Detection
Source: Microorganisms. 2025 Sep 26;13(10):2259. doi: 10.3390/microorganisms13102259 (PMC12566355; doi:10.3390/microorganisms13102259)
Supplement: Supplementary file 1 [file microorganisms-13-02259-s001.zip › microorganisms-3828784-supplementary.pdf]

Table S1. This table presents the LOD values obtained for three Enterobacterales strains (*E. coli*, *K. pneumoniae*, and *S. ureilytica*) across four different culture-based methods. For each method, limit of detection was assessed using three independent biological replicates on separate days, each with two technical replicates. Each technical replicate was plated on two agar plates, and the mean colony count from these plates was used to determine the limit of detection (CFU/mL), as shown in the corresponding column.

Culture-based method	Species	Biological replicate /technical replicate	Limit of detection (CFU/mL)
Method 1	*E. coli* ATCC 25922	1/1	$4{,}95*10^2$
	E. coli ATCC 25922	1/2	$4{,}95*10^2$
	E. coli ATCC 25922	2/1	$1{,}03*10^3$
	E. coli ATCC 25922	2/2	$1{,}03*10^2$
	E. coli ATCC 25922	3/1	$2{,}7*10^1$
	E. coli ATCC 25922	3/2	$2{,}7*10^2$
	K. pneumoniae ATCC 700603	1/1	$4{,}27*10^2$
	K. pneumoniae ATCC 700603	1/2	$4{,}27*10^2$
	K. pneumoniae ATCC 700603	2/1	$4{,}0*10^1$
	K. pneumoniae ATCC 700603	2/2	$4{,}0*10^1$
	K. pneumoniae ATCC 700603	3/1	$7{,}2*10^1$
	K. pneumoniae ATCC 700603	3/2	$7{,}20*10^2$
	S. ureilytica S.35.25.Sm	1/1	$1{,}49*10^2$
	S. ureilytica S.35.25.Sm	1/2	$1{,}49*10^2$
	S. ureilytica S.35.25.Sm	2/1	$2{,}17*10^2$
	S. ureilytica S.35.25.Sm	2/2	$2{,}17*10^2$
	S. ureilytica S.35.25.Sm	3/1	$1{,}33*10^2$
	S. ureilytica S.35.25.Sm	3/2	$1{,}33*10^2$
Method 2	*E. coli* ATCC 25922	1/1	$4{,}95*10^1$
	E. coli ATCC 25922	1/2	$4{,}95*10^1$
	E. coli ATCC 25922	1/3	$4{,}95*10^1$
	E. coli ATCC 25922	2/1	$1{,}09*10^1$
	E. coli ATCC 25922	2/2	$1{,}09*10^1$
	E. coli ATCC 25922	2/3	$1{,}09*10^2$
	E. coli ATCC 25922	3/1	2.7
	E. coli ATCC 25922	3/2	$2{,}7*10^1$
	E. coli ATCC 25922	3/3	$2{,}7*10^1$
	K. pneumoniae ATCC 700603	1/1	4
	K. pneumoniae ATCC 700603	1/2	4
	K. pneumoniae ATCC 700603	1/3	$4{,}0*10^1$
	K. pneumoniae ATCC 700603	2/1	7,15
	K. pneumoniae ATCC 700603	2/2	7,15
	K. pneumoniae ATCC 700603	2/3	7,15
	K. pneumoniae ATCC 700603	3/1	6,47
	K. pneumoniae ATCC 700603	3/2	$6{,}47*10^1$
	K. pneumoniae ATCC 700603	3/3	$6{,}47*10^1$
	S. ureilytica S.35.25.Sm	1/1	$2{,}17*10^1$
	S. ureilytica S.35.25.Sm	1/2	$2{,}17*10^1$
	S. ureilytica S.35.25.Sm	1/3	2,17
	S. ureilytica S.35.25.Sm	2/1	$1{,}32*10^1$

	S. ureilytica S.35.25.Sm	2/2	1,32*10^{1}
	S. ureilytica S.35.25.Sm	2/3	1,32
	S. ureilytica S.35.25.Sm	3/1	1,19*10^{1}
	S. ureilytica S.35.25.Sm	3/2	1,19*10^{1}
	S. ureilytica S.35.25.Sm	3/3	1.19*10^{2}
Method 3	*E. coli* ATCC 25922	1/1	1,02
	E. coli ATCC 25922	1/2	1,02*10^{1}
	E. coli ATCC 25922	2/1	2,25*10^{1}
	E. coli ATCC 25922	2/2	2,25
	E. coli ATCC 25922	3/1	1,62
	E. coli ATCC 25922	3/2	1,62
	K. pneumoniae ATCC 700603	1/1	1,78
	K. pneumoniae ATCC 700603	1/2	1,78
	K. pneumoniae ATCC 700603	2/1	1,78
	K. pneumoniae ATCC 700603	2/2	1,78*10^{1}
	K. pneumoniae ATCC 700603	3/1	2,1
	K. pneumoniae ATCC 700603	3/2	2,1
	S. ureilytica S.35.25.Sm	1/1	1,58
	S. ureilytica S.35.25.Sm	1/2	1,58
	S. ureilytica S.35.25.Sm	2/1	2,41*10^{1}
	S. ureilytica S.35.25.Sm	2/2	2,41*10^{1}
	S. ureilytica S.35.25.Sm	3/1	1,62
	S. ureilytica S.35.25.Sm	3/2	1,62
Method 4	*E. coli* ATCC 25922	1/1	1,75
	E. coli ATCC 25922	1/2	3,75
	E. coli ATCC 25922	2/1	5,5
	E. coli ATCC 25922	2/2	5,5
	E. coli ATCC 25922	3/1	5,22
	E. coli ATCC 25922	3/2	6,12
	K. pneumoniae ATCC 700603	1/1	9,5
	K. pneumoniae ATCC 700603	1/2	9,82
	K. pneumoniae ATCC 700603	2/1	7,04
	K. pneumoniae ATCC 700603	2/2	7,45
	K. pneumoniae ATCC 700603	3/1	3,85
	K. pneumoniae ATCC 700603	3/2	5,17
	S. ureilytica S.35.25.Sm	1/1	2,01
	S. ureilytica S.35.25.Sm	1/2	1,99
	S. ureilytica S.35.25.Sm	2/1	1,68
	S. ureilytica S.35.25.Sm	2/2	7,9
	S. ureilytica S.35.25.Sm	3/1	1,56
	S. ureilytica S.35.25.Sm	3/2	1,65

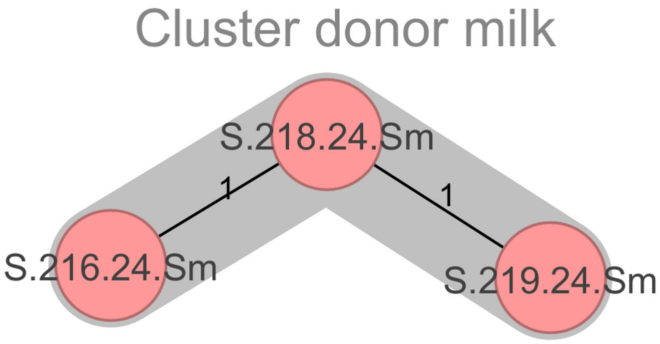

Figure S1. Minimum Spanning Tree illustrating the clonal relationship of three *Serratia ureilytica* isolates (S.216.24.Sm, S.218.24.Sm, S.219.24.Sm) recovered from raw donor milk samples in January 2024. The numbers indicate the allelic differences based on core genome MLST (cgMLST), with all isolates differing by only one allele, indicating clonal relatedness.
